# Sorghum aphid/greenbug: current research and control strategies to accelerate the breeding of aphid-resistant sorghum

**DOI:** 10.3389/fpls.2025.1588702

**Published:** 2025-06-03

**Authors:** Zhiyin Jiao, Jinping Wang, Xue Ma, Yannan Shi, Zhifang Wang, Yongchao Guo, Peng Lv

**Affiliations:** Institute of Millet Crops, Hebei Academy of Agriculture and Forestry Sciences, Hebei Branch of China National Sorghum Improvement Center, Shijiazhuang, China

**Keywords:** sorghum, sorghum aphid, greenbug, aphid resistance mechanisms, QTL mapping, molecular breeding, chemical and biological control, food security

## Abstract

Sorghum, one of the world’s five major cereal crops, faces significant yield losses due to aphid infestations, particularly from the sorghum aphid (*Melanaphis sacchari*) and the greenbug (*Schizaphis graminum*). These pests not only cause a reduction in grain yield, but also transmit plant viruses, posing a serious threat to global food security. Current strategies to mitigate aphid damage include large-scale insecticide applications, biological control through natural enemies, and the development of aphid-resistant sorghum varieties. However, the resistance genes of aphids and their mechanisms are still unclear, which poses a major challenge to breeding programs. This review synthesizes recent advances in understanding the interactions between sorghum and these two major aphid species, exploring topics such as aphid classification, quantitative trait locus (QTL) mapping of resistance genes, and the molecular mechanisms of sorghum-aphid interactions. We also discuss conventional and emerging insecticide methods, biological control strategies, and their associated challenges. Looking ahead, the integration of molecular breeding techniques, including genetic engineering and genome editing, holds promise for accelerating the development of aphid-resistant sorghum varieties. These innovative approaches aim to minimize aphid damage, enhance sorghum productivity, and contribute to global food security in the face of climate change and evolving pest pressures.

## 1 Introduction

Sorghum [*Sorghum bicolor* (L.) Moench], originating from sub-Saharan Africa, is the world’s fifth largest cereal crop, following wheat, rice, maize, and barley, which is widely cultivated globally (Brown et al., 2006; Takanashi, 2023). Global sorghum production is approximately 61 million tons every year, serving as a staple food for more than 500 million people in 30 countries across Africa and Asia. In China, the annual production is around 3 million tons (Khoddami et al., 2023). As a C_4_ model crop, sorghum is different from other cereals and can be grown in extreme environments under abiotic stresses such as drought and heat (Jeff and Dahlberg, 2019; Rudo et al., 2021). Facing the challenges of a growing global population and intensifying global climate change, sorghum as a resilient crop, plays a key role in feeding more impoverished people and safeguarding global food security (Al-Salman et al., 2024). Despite its advantages in resisting abiotic stresses, sorghum faces severe challenges from biotic stresses (Erpen et al., 2018; Baillo et al., 2019; Min et al., 2023; Zhang et al., 2023). During its growth period, sorghum is susceptible to over 150 pests (Kruger et al., 2008; Guo et al., 2011). With the global expansion of sorghum cultivation, aphid (*Acyrthosiphon pisum*) infestations have become a significant issue in regions such as China, North America, and South Africa, posing a major threat to sorghum production (Singh et al., 2004; Bowling et al., 2017; Craigie et al., 2024). There are about 5500 species of aphids, with approximately 250 species considered economically significant pests that can cause serious harm to plants (Powell, 1992; Blackman and Eastop, 2006; Isaacs and Woodford, 2008). Among them, the sorghum aphid [*Melanaphis sacchari* (Zehntner)], the greenbug [*Schizaphis graminum* (Rondani)], the corn leaf aphids [*Rhopalosiphum maidis* (Fitch)], and the bird cherry-oat aphid [*Rhopalosiphum padi* (Linnaeus)] are the four main aphid species that cause significant damage to sorghum production (Kariyat et al., 2019; Lopes et al., 2022; Carl et al., 2024; Zhao et al., 2024a). Two of those four species, the sorghum aphid and the greenbug have a profound impact on sorghum yield and cause serious damage (Zhao et al., 2024b). Among them, sorghum aphid can reduce sorghum yield by 50% to 100% (Thudi et al., 2024).

The sorghum aphid, a member of the Homoptera: Aphididae family, has a complex taxonomic history. The sugarcane aphid (*Melanaphis sacchari*) was first identified on sugarcane in Java, Indonesia, in 1897, while the sorghum aphid (*Melanaphis sorghi*) was initially discovered on sorghum in Sudan in 1904. Although these two species have often been considered synonymous, recent studies suggest distinct host preferences: the sugarcane aphid primarily infests sugarcane, whereas the sorghum aphid is more commonly associated with sorghum (Paudyal et al., 2019). Nibouche et al. (2021) analyzed several aphid samples from the United States and identified morphological differences between sorghum aphids and sugarcane aphids (Nibouche et al., 2021). This research provides preliminary evidence supporting the existence of distinct differences between the two species. However, recent studies have revealed that sugarcane aphids can also experience large outbreaks in sorghum fields, this finding suggests that the distinction between the two feeding species is not well-defined (Paudyal et al., 2019). Therefore, researchers still tend to categorize the two species as a single species, named the sorghum aphid (*M. sorghi*).

The sorghum aphid severely impacts global sorghum yields by feeding on the sap within the phloem of stems and leaves throughout all growth stages of the plant (Xoconostle-Cázares et al., 2023). While feeding on sap in the phloem, the sorghum aphid can secrete honeydew, which can reduce plant photosynthesis and affects metabolic reactions (Vasquez et al., 2024). Furthermore, sorghum aphids can transmit various plant viruses, such as the cereal red leaf virus, sugarcane yellow leaf virus, and sugarcane mosaic virus (Kondaiah and Nayudu, 1984; Gupta and Virendra, 1994; Schenck and Lehrer, 2000). These viruses can lead to significant losses in sorghum production and quality, compromising the safety of food and feed, and posing risks to the health of both humans and animals (Bowling et al., 2017; Shabbir et al., 2022). Sorghum aphids, with their high reproductive capacity, can quickly disperse by flight when encountering resistance. Traditional chemical control methods, while effective, are labor-intensive, time-consuming, and pose risks of environmental pollution, making complete eradication of aphids a persistent challenge (Pekarcik and Jacobson, 2021).

The greenbug (Hemiptera: Aphididae), was first reported in 1907 (Webster, 1907). As well as the sorghum aphid, it is one of the harmful aphid species that reduce global sorghum production (Bonnie et al., 2009; Zhang and Huang, 2024). Based on the different host and plant responses to greenbug, they can be categorized as A, B, C, E, F, G, H, I, J, K, Chn1, NY, WY10MC, WY81, WY10 B, WY12 MC, and WY86 (Royer et al., 2015). The greenbug uses its needle-like mouthparts to pierce the plant’s phloem, extracting sap while injecting toxic saliva into the plant. This dual action causes significant damage to sorghum, impacting its growth and productivity (Burton and Burd, 1993; Bonnie et al., 2009; Xu et al., 2024). After feeding on sorghum, greenbugs cause the leaves to develop red spots, which gradually turn yellow and eventually lead to the death of the affected tissue (Teetes and Johnson, 1974). In one study, the result indicates that after feeding by Biotype C greenbugs, the organelle recognition function of nearby phloem cells is disrupted. Chloroplast membranes are damaged, and mitochondria undergo gradual degeneration, resulting in severe structural and functional impairment of the organelles (Al-Mousawi et al., 1983). With a strong reproductive capacity, a single female greenbug can produce 60–80 offspring and the population of greenbug can double every two days under ideal conditions (Royer et al., 2015). Therefore, finding efficient ways to control greenbug is crucial for mitigating their impact on global sorghum production in the future.

In agricultural management, it is common to use insecticides to address and prevent aphid infestations. While the large-scale application of insecticides can yield the desired results, it also poses several risks. Firstly, the excessive use of insecticides can lead to genetic mutations in aphids, allowing them to develop resistance to these chemicals. Moreover, due to their rapid reproduction rate, the misuse of insecticides may further accelerate the mutation rate of aphids, making it increasingly challenging to control new aphid populations. Additionally, the widespread use of potentially hazardous insecticides contradicts our current principles of environmental protection. Therefore, it is essential to explore alternative methods for aphid control to achieve a more sustainable approach to agricultural production. Sorghum has a relatively small genome (730M) that has been sequenced and can be accessed through online databases like the National Center for Biotechnology Information (NCBI, https://www.ncbi.nlm.nih.gov) and Phytozome (https://phytozome-next.jgi.doe.gov). These databases are of significant importance for identifying molecular markers associated with aphid-related genes and their practical applications in agriculture (Goodstein et al., 2012; Agarwala et al., 2018; Rudo et al., 2021). Leveraging diverse sorghum germplasm resources, identifying key aphid resistance genes, and unraveling the molecular mechanisms of sorghum resistance are essential steps to accelerate the development of resistant sorghum varieties and revitalize the sorghum seed industry. This review synthesizes prior research on the classification of sorghum aphids and greenbugs, plant responses to aphid feeding, and the identification of plant resistance genes against aphids. It also highlights future directions in sorghum breeding for aphid resistance, emphasizing the strategic use of molecular markers and advanced tools to accelerate the discovery of resistance genes and develop high-yielding, aphid-resistant sorghum varieties-a critical challenge for the future.

## 2 Aphid resistance materials

Previous studies have indicated a scarcity of aphid-resistant sorghum germplasm resources. Based on the survival rate of seedlings after aphid treatment, Singh et al. (2004) reported 18 sorghum germplasm resources with high resistance from Ethiopia, Congo, Malawi, the United States, Mexico, India, and Japan (Singh et al., 2004). Lu and Dahlberg (2001) identified approximately 5,000 germplasm resources from China, with only 1 material showing high resistance to aphid, which was homologous to the US sorghum TAM428 (Lu and Dahlberg, 2001). In recent years, several studies have conducted aphid resistance identification on more sorghum materials, the results showed that through the assays by using the nylon net, clip cage and leaf disc, 10 with moderate resistance and 6 with high resistance were identified. Another approach was to evaluate the aphid damage level by using plant height, the number of leaves and chlorophyll loss, and 2 materials with high tolerance were identified (Sharma et al., 2014; Mbulwe et al., 2016; Paudyal et al., 2019). Information on germplasm resources and sorghum materials exhibiting high resistance is summarized in **Table 1**. Knoll et al. (2023) identified three aphid-resistant sweet sorghum varieties, named GTS1903, GTS1904, and GTS1905, through field selection and trait observation (Knoll et al., 2023). These varieties share a common origin, all being derived from the resistant parent PI 257599, which carries known resistance loci on SBI-06, as confirmed through genetic marker identification. Huang and Huang (2023) also discovered the PI550607 line, which possessed resistance to greenbug (Huang and Huang, 2023). Guden et al. (2019) screened 561 sorghum materials and identified 26 sweet sorghum varieties with excellent agronomic traits. Through field trials and aphid population statistics, they ultimately obtained the aphid-resistant sweet sorghum variety BSS507, which showed high resistance at two experimental sites (Guden et al., 2019). In addition, previous studies identified aphid resistant/tolerant sorghum lines, including IS1144C, IS12664C, IS12609C, and TAM428 as resistant lines (Singh et al., 2004), while TX3408, TX3409 (Mbulwe et al., 2016), AG1201, AG1301, W844-E, and DKS37-07 (Paudyal et al., 2019) are tolerant lines. It is important to note that the origin and mechanisms of resistance or tolerance among these materials remain unclear and require further investigation. These sorghum lines exhibiting resistance to aphids hold potential for future breeding development as restorer lines (Restorer line is used to restore the fertility in hybrid after crossing with ms A line in hybrid production plot).

**Table 1:** Sorghum materials resistant/tolerance to aphids.

Germplasm/materials	Origin	Resistance/Tolerance	Reference
IS1133C	India	HR	Teetes et al., 1995
IS1134C	India	HR	Teetes et al., 1995
IS1139C	India	HR	Teetes et al., 1995
IS1144C	India	HR	Teetes et al., 1995
IS1598C	India	HR	Teetes et al., 1995
IS5188C	India	HR	Teetes et al., 1995
IS5887C	India	HR	Teetes et al., 1995
IS6389C	India	HR	Teetes et al., 1995
IS6416C	India	HR	Teetes et al., 1995
IS6426C	India	HR	Teetes et al., 1995
IS8100C	Japan	HR	Teetes et al., 1995
IS12158C	Ethiopia	HR	Teetes et al., 1995
IS12551C	Ethiopia	HR	Teetes et al., 1995
IS12599C	Congo	HR	Teetes et al., 1995
IS12608C	Ethiopia	HR	Teetes et al., 1995
IS12645C	Ethiopia	HR	Teetes et al., 1995
IS12661C	Ethiopia	HR	Teetes et al., 1995
IS12664C	Ethiopia	HR	Teetes et al., 1995
Tx3408	America	HT	Mbulwe et al., 2016
Tx3409	America	HT	Mbulwe et al., 2016
AG1201	America	HR	Paudyal et al., 2019
AG1203	America	HR	Paudyal et al., 2019
W844-E	America	HR	Paudyal et al., 2019
DKS37-07	America	HR	Paudyal et al., 2019
H13073	America	HR	Paudyal et al., 2019
GW1489	America	HR/HT	Paudyal et al., 2019

HR, high resistance; HT, high tolerance.

## 3 Aphids genotypes

### 3.1 Sorghum aphid genotypes

**Figure 1:**
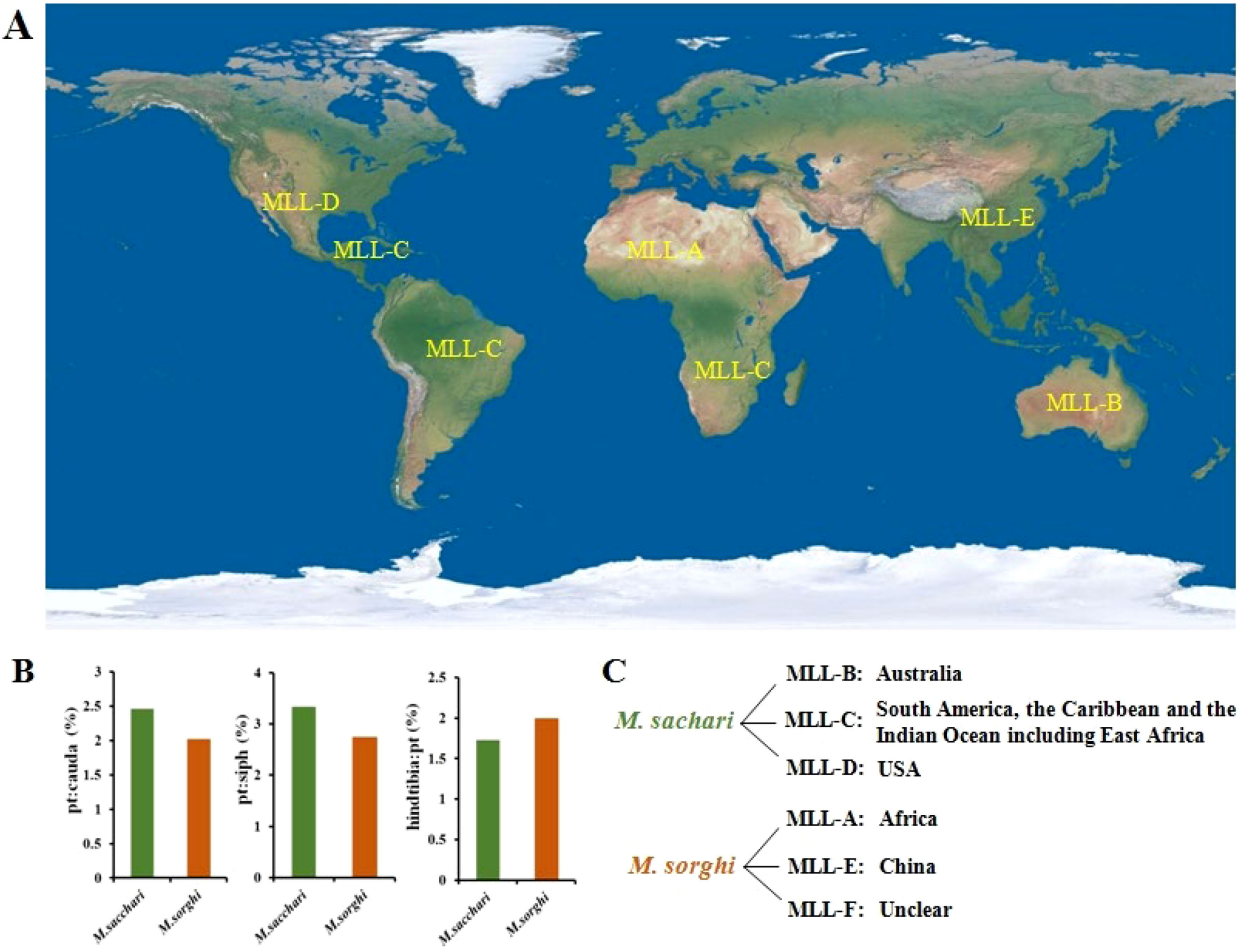
Lineage classification and origin of sorghum aphids. **(A)** Global origin distribution of six sorghum aphid lineages. **(B)** Differences in pt:cauda, pt:siph, and hindtibia:pt between sorghum aphids and sugarcane aphids. **(C)** Different lineages and origins of sorghum aphids and sugarcane aphids. pt, processus terminalis length; siph, siphunculi (Nibouche et al., 2021).

Sorghum aphid (*M. sacchari*) was first studied in terms of its classification in 2014 by Nibouche. They conducted genetic typing of aphids by analyzing 1333 individuals collected from sugarcane and sorghum between 2002 and 2009. They defined five multi-locus lineages (MLL) for *M. sacchari* based on their origin: MLL-A, Africa; MLL-B, Australia; MLL-C, South America, the Caribbean, and the Indian Ocean (including East Africa); MLL-D, USA; MLL-E, China (Nibouche et al., 2014); Following outbreaks of aphids in the United States and Caribbean coastal countries, Nibouche et al. (2018) identified a new clone lineage through satellite markers and sequencing, named MLL-F. MLL-F has been found to spread extensively on both sugarcane and sorghum. As an invasive species in the USA, its origin remains unclear. While *M. sorghi* and *M. sacchari* have traditionally been considered synonymous, experimental evidence has not definitively established whether they are the same species. Different lineages infect different host plants, hinting at potential distinctions between the two types (Nibouche et al., 2018). Further, they analyzed 199 aphid samples collected over 14 years in the USA using morphometrics and molecular data. They concluded that MLL-B, MLL-C, and MLL-D belong to *M. sacchari*, while MLL-A, MLL-E, and MLL-F belong to *M. sorghi*. Morphological differences were observed in features such as the length of the cauda, hindtibia, siphunculi, and processus terminalis length between *M. sacchari* and *M. sorghi* (Nibouche et al., 2021). In 2022, the sorghum aphid crisis on sorghum in Brazil was confirmed to be MLL-F, the same lineage as the 2013 outbreak in the USA (Harris-Shultz et al., 2022b). In this article, the mentioned *M. sacchari* already includes *M. sorghi*, which are considered synonymous. Although these has been divided into six lineages, it remains one of the aphid species with the least known genetic diversity within such a widespread global distribution of aphids (**Figure 1**).

### 3.2 Greenbug genotypes

The greenbug (Hemiptera: Aphididae) is a major pest that threatens sorghum crops worldwide. Research on this pest began in 1961, initially focusing on classifying different types based on the plants’ reactions to greenbug infestations (Wood, 1961; Shufran et al., 2010). Wood isolated a biotype of greenbug from wheat that could harm susceptible varieties but could not grow on resistant ones, referred to as the A biotype. Further research identified a new greenbug that could damage A biotype resistant wheat, which was named the B biotype. Harvey and Hackerott (1969) isolated a new strain, the C biotype, from severely affected sorghum populations, exhibiting different sensitivities to various plants compared to the previously identified B biotype from wheat (Harvey and Hackerott, 1969). Cress and Chada (1971) found metabolic differences between the A and B biotypes, with the A biotype having a slower metabolism (Cress and Chada, 1971). Porter et al. (1982) identified a different resistance biotype, the E biotype, in wheat populations (Porter et al., 1982). Beregovoy et al. (1988) analyzed sorghum populations affected by greenbug biotype C and E in eight US regions, highlighting the E biotype higher reproduction on oats and Sudan grass but shorter survival on maize (Beregovoy et al., 1988). Kindler and Spomer (1986) discovered a new biotype, the F biotype, closely related to the A biotype in plant response, capable of killing ‘Reubens’ Canada bluegrass, L., resistant to biotypes A-E (Kindler and Spomer, 1986). Puterka et al. (1988) obtained two new strains, the G and H biotypes, from host plants in Oklahoma (SCO) and Texas (WCT) (Puterka et al., 1988). Harvey et al. (1991) isolated a severe biotype, the I biotype, from hybrid sorghum resistant to the E biotype (Harvey et al., 1991). Beregovoy and Peters (1994) identified the J biotype in a barley variety, POST, sensitive to barley but harmless to resistant wheat (Beregovoy and Peters, 1994). Harvey et al. (1997) separated the K biotype from the sorghum line PI550610 (resistant to the I biotype), with the potential for breeding due to high resistance. In addition to the A-K biotypes, several other biotypes have been reported (Harvey et al., 1997). Liu and Jin (1998) identified and named a new biotype, Chn1, on wheat varieties in Beijing, which poses significant harm to wheat but not to oats or rye (Liu and Jin, 1998). Shufran et al. (2010) isolated a new biotype, NY, from *Elymus canadensis* (L.) (Shufran et al., 1997). Armstrong et al. (2016) identified five new biotypes from wild barley varieties, namely WY10 MC, WY81, WY10 B, WY12 MC, and WY86. In total, there are currently 17 biotypes (**Table 2**), with C, E, I, and K types significantly impacting sorghum yield (Armstrong et al., 2016). Understanding the differences in resistance of various sorghum varieties to different biotypes of the greenbug is crucial for the application of resistance genes in breeding efforts.

**Table 2:** Biotype classification of the greenbug.

Biotype	Host collected	Reference
A	Wheat	Wood, 1961
B	Wheat	Wood, 1961
C	Sorghum	Harvey and Hackerott, 1969
E	Wheat	Porter et al., 1982
F	Canada bluegrass	Kindler and Spomer, 1986
G	Wheat	Puterka et al., 1988
H	Wheat	Puterka et al., 1988
I	Sorghum	Harvey et al., 1991
J	Wheat	Beregovoy and Peters, 1994
K	Sorghum	Harvey et al., 1997
Chn1	Wheat	Liu and Dasheng, 1998
NY	*Elymus canadensis*	Shufran et al., 2010
WY10 MC	Barley	Armstrong et al., 2016
WY81	Barley	Armstrong et al., 2016
WY10 B	Barley	Armstrong et al., 2016
WY12 MC	Barley	Armstrong et al., 2016
WY86	Barley	Armstrong et al., 2016

## 4 Progress in molecular mechanisms of aphid resistance in sorghum

In the following section, we provide a detailed overview of recent research advancements related to sorghum aphids and greenbugs, focusing on aphid classification, plant resistance genes against aphids, and plant responses to aphid infestation.

### 4.1 Molecular mechanisms of sorghum resistance to sorghum aphid

#### 4.1.1 Genetic studies of sorghum aphid resistant genes and QTLs

Currently, the candidate genes related to aphid resistance in sorghum and the molecular responses of sorghum to aphid infestation are not fully understood. Chang (1981) demonstrated that sorghum resistance to sorghum aphids is controlled by a single dominant gene (Chang, 1981). This conclusion was also supported by crossing the high aphid resistance sorghum variety HN-16 with QS, the aphid resistance gene mapping study was conducted for the F1 and F2 generations (Chang et al., 2006). through experiments. In recent years, an increasing number of molecular markers closely linked to aphid resistance genes have been developed. Wang et al. (2013) utilized two molecular markers, Sb6m2650 and Sb6rj2776, to map a candidate region for the sorghum resistance gene *RMES1* (*Resistance to M. sacchari 1*) on the short arm of chromosome 6, spanning 126kb and containing 5 candidate genes, namely *Sb06g001620 (Sobic.006G017000)*, *Sb06g001630 (Sobic.006G017100)*, *Sb06g001640 (Sobic.006G017200)*, *Sb06g001645 (Sobic.006G017400)* and *Sb06g001650 (Sobic.006G017500)* (Wang et al., 2013). Wang et al. (2021) using Tx2783 as a reference, combined with materials such as BTx623, RTx430, and Rio, a feature analysis was conducted on structural variations (SVs) including insertion (INS), delivery (DEL), inversion (INV), and copy number variation (CNV) among different varieties, and the same region was determined (Wang et al., 2021). Zhang et al. (2021) identified four QTLs from the sorghum aphid-resistant resource 407B, one of which, qtlMs-6.1, falls within the candidate region of the *RMES1* gene (Zhang et al., 2021). Muleta et al. (2022) analyzed the gene sequences of the five candidate genes in the 126kb region using the aphid-resistant line PI276837 and three aphid-susceptible lines, BTx623, Tx430, and BTx642. They found 35, 32, and 29 SNP variations in the exons of *Sobic.006G017200*, *Sobic.006G017400*, and *Sobic.006G017500*, respectively, and 3 insertions/deletions in *Sobic.006G017500* (Muleta et al., 2022). Punnuri et al. (2022) reported five markers associated with plant response to sorghum aphids, two of which were related to aphid numbers and sorghum damage, located on chromosome 8 (*S8_11781182*) and chromosome 10 (*S10_2507813*), and three were related to sorghum damage on chromosomes 2 (*S2_61431704*), 3 (*S3_19558428*), and 5 (*S5_63115845*) (Punnuri et al., 2022). The known markers and QTLs are summarized in **Table 3**.

**Table 3:** Markers/QTLs loci identified for sorghum aphid resistance.

Marker/QTL	Location	Parental lines	annotation	Reference
Sb6m2650- Sb6rj2776	6	HN16×BTx623	126kb, including RMES1	Wang et al., 2013
qtlMs-6.1	6	407B×7B	located in Sobic.006G017200	Zhang et al., 2021
qtlMs-6.2	6	407B×7B		Zhang et al., 2021
qtlMs-6.3	6	407B×7B		Zhang et al., 2021
qtlMs-6.4	6	407B×7B		Zhang et al., 2021
S2_61431704	2	The Sorghum Association Panel(SAP)	close to an Avr9 elicitor response protein	Punnuri et al., 2022
S3_19558428	3	The Sorghum Association Panel(SAP)		Punnuri et al., 2022
S5_63115845	5	The Sorghum Association Panel(SAP)	an oxidative stress response gene similar to Cytochrome P450 71E1	Punnuri et al., 2022
S8_11781182	8	The Sorghum Association Panel(SAP)	similar to Diacylglycerol kinase 1, as they were narrowed down to single genes from the previous gene expression studies around these markers	Punnuri et al., 2022
S10_2507813	10	The Sorghum Association Panel(SAP)	Among 32 genes, including several LRR repeats	Punnuri et al., 2022

In recent years, with the completion of sorghum genome sequencing and the continuous development of technologies such as transcriptomics and proteomics, research on the aphid resistance mechanism of sorghum has been progressing gradually. Through transcriptomic and metabolomic analyses of the aphid resistant sorghum variety HN-16 and the aphid sensitive variety BTx623, it was shown that the differentially expressed genes were mainly enriched in the flavonoid biosynthesis pathway, and the differentially expressed metabolites were mainly related to isoflavone biosynthesis and flavonoid biosynthesis. Furthermore, the observation of the epidermal cell structures of two different varieties revealed that the resistance of sorghum to aphids is positively correlated with the regularity of epidermal cells and negatively correlated with cell spacing and leaf thickness (Zhao et al., 2024b). Tetreault et al. (2019) demonstrated that jasmonic acid (JA), ethylene (ETH), and other plant hormones can regulate a plant’s resistance to sorghum aphids by comparing transcriptome results of aphid-resistant and susceptible materials under sorghum aphid stress (Tetreault et al., 2019). Grover et al. (2024) discovered that Auxin-Aspartic Acid (IAA-Asp) can enhance plant resistance to aphids in sorghum *Brown midrib* (*Bmr*) mutants, and *Bmr* negatively regulates IAA-Asp content (Grover et al., 2024). Subsequently, Grover et al. (2022b) conducted a proteomic analysis on the aphid-resistant genotype SC265, which was determined as a resistant line through choice and no-choice assays, revealing an upregulation of defense and signal-related proteins after 1 and 7 days of aphid feeding, including salicylic acid (SA), phospholipase, calcium signaling, and Zinc-related proteins (Grover et al., 2022b). Furthermore, studies suggested that ARFs (Auxin Response Factors), GRAS, MADS, NAC (NAM, ATAF1/2, CUC1/2), and WRKY transcription factor families are involved in sorghum’s response to aphids (Serba et al., 2021). Poosapati et al. (2022) demonstrated that overexpression of *SbWRKY86* in *tobacco* and *Arabidopsis* can increase resistance to peach aphids (*Myzus persicae*), but did not directly prove resistance to sorghum aphids (Poosapati et al., 2022). Therefore, it can be a potential candidate gene for the resistance of sorghum aphids, and more in-depth studies are needed to understand its response pattern to sorghum aphids. The nucleotide-binding site (NBS)-leucine-rich repeat (LRR) gene family is an important plant disease resistance gene, widely present in plants, animals, and fungi, commonly involved in defense response signal transduction (Balamurugan et al., 2024). Tetreault et al. (2019) found that over 70 LRR genes were upregulated in aphid-resistant sorghum lines through transcriptome analysis, indicating that LRR proteins confer resistance to aphids in sorghum (Tetreault et al., 2019). Among the five candidate genes identified by Wang et al. (2013), three genes are LRR genes (Wang et al., 2013). Subsequent research can focus on the NBS-LRR gene family to further elucidate the function of each candidate gene.

#### 4.1.2 Sorghum defensive response to sorghum aphid infestation

Aphids feeding can trigger the host plants to respond to the defense signaling pathways (Thudi et al., 2024). In this section, we briefly summarized the morphological and structural changes and hormone signals of sorghum after sorghum aphid infection. After being attacked by sorghum aphids, sorghum triggers a series of signal transductions internally to initiate plant defense responses. This intricate biological process involves multiple signaling pathways, including the coordinated action of hundreds of genes, various plant hormones, secondary metabolites, and other compounds that collectively respond to aphids (Smith and Boyko, 2010). Initially, plants undergo physiological changes in response to aphid feeding, such as forming cuticle layers and epicuticular waxes on leaves (Harris-Shultz et al., 2022b; Cardona et al., 2023b). To study the impact of epicuticular waxes on aphid feeding, Cardona et al. (2023a, b) used electrical penetration graph technology to detect aphid feeding process. They found that aphids tend to feed longer from phloem and subsequent experiments have shown that aphids prefer to feed on non-flowering plants with high wax content. In non-flowering plants, higher levels of 16-monoacylglycerol and c32-alcohol suggest these substances may influence aphid feeding on sorghum. This highlights the crucial role of epicuticular waxes in plant resistance against aphid feeding (Cardona et al., 2023a, b). Triplett et al. (2023) discovered that stomatal density, trichome density, and chloroplast density show a positive correlation with aphid resistance, whereas trichome length is negatively correlated with aphid resistance (Triplett et al., 2023). By comprehensively understanding these relationships, we can facilitate the breeding of plant varieties that possess greater resistance to aphids, ultimately leading to improved crop productivity and sustainability. Grover’s research found that plant hormones like JA and cytokinins (CTK) play essential roles in sorghum’s defense against aphids (Grover et al., 2022a). Additionally, plants produce volatile chemicals like alkaloids and sorghum ketone to defend against aphids (Mizuno et al., 2010). Some studies suggested that sorghum can reduce aphid populations by prolonging the presence of aphid predators. Sorghum serves as a food source for aphid predators like hoverflies and bees, which also collect honeydew produced by aphids. Planting susceptible sorghum varieties around the edges of fields may help defend against aphids effectively (Harris-Shultz et al., 2022a).

### 4.2 Molecular mechanisms of sorghum resistance to greenbug

#### 4.2.1 Genetic studies of greenbug resistant genes and QTLs

Research on the genetic mapping of the greenbug resistance can be traced back to 2002, when Katsar et al. (2002) reported 9 QTL loci associated with sorghum resistance to the greenbug. These QTLs are located on the following chromosomes: chr1 (*Ssg4*, bio: E), chr4 (*Ssg7*, bio: K), chr5 (*Ssg2*, bio: E/C; *Ssg9*, bio: E), chr6 (*Ssg5*, bio: E), chr7 (*Ssg1*, bio: E), chr8 (*Ssg3*, bio: K), chr9 (*Ssg6*, bio: K), and chr10 (*Ssg8*, bio: I). Notably, Ssg2 and Ssg9 can explain approximately ≈30% of the variation in the resistance phenotype, respectively (Katsar et al., 2002). Wu and Huang (2008) reported two QTL loci associated with greenbug resistance, both located on chr9 (*QSsgr-09-01*, PVE: 54.5-80.3%, bio: I; *QSsgr-09-02*, PVE: 1.3-5.9%, bio: I), from the cross Westland A×PI550610 through the construction of an F2:3 population (Wu and Huang, 2008). Next, Punnuri et al. (2013) identified four QTL loci associated with resistance to greenbug with a biotype I through the construction of an F2 population from the cross PI 607900×BTx623. These QTL, named *Qstsgr-sbi09i*, *Qstsgr-sbi09ii*, *Qstsgr-sbi09iii*, and *Qstsgr-sbi09iv*, are all located on chr9 and collectively explain 17.3-82.4% of the phenotypic variation (Punnuri et al., 2013). Subsequently, Punnuri and Huang (2017) conducted QTL mapping again using two parental lines, BTx623 (susceptible) and PI 607900 (resistant), and obtained consistent QTL analysis results with those identified using the F2 population in the previous study (Punnuri and Huang, 2017). Nagaraj et al. (2005) identified 6 QTLs using recombinant inbred lines (RILs) derived from the cross between ‘96-4121’ (resistant) and Redlan (susceptible). These QTLs were located on chr4 (*VIS-GBK1*, bio: K; *VIS-GBK2*, bio: K; *VIS-GBI8*, bio: I) and chr5 (*VIS-GBK5*, bio: K; *SPA-I2*, bio: I; *SPA-K2*, bio: K), these QTLs can explain phenotypic variation ranging from 9% to 19.6% (Nagaraj et al., 2005). The known QTLs are summarized in **Table 4**.

**Table 4:** QTL loci identified for greenbug resistance.

QTL	Position	Biotype	Origin/parental lines	LOD value	Reference
*Ssg1*	7(127)	E	SA7536-1、Capbam、PI 550607		Katsar et al., 2002
*Ssg2*	5(60)	E/C	SA7536-1、Capbam		Katsar et al., 2002
*Ssg3*	8(69-101)	K	BTx623、PI 550607		Katsar et al., 2002
*Ssg4*	1(108)	E	BTx623、PI 550607		Katsar et al., 2002
*Ssg5*	6(78)	E	BTx623、PI 550607		Katsar et al., 2002
*Ssg6*	9(73)	K	Capbam、PI550607		Katsar et al., 2002
*Ssg7*	4(70)	K	PI550607		Katsar et al., 2002
*Ssg8*	10(102)	I	PI550607		Katsar et al., 2002
*Ssg9*	5(18)	E	Capbam		Katsar et al., 2002
*QSsgr-09-01*	9(9.3)	I	Westland A×PI550610	14.2-39.5	Wu and Huang, 2008
*QSsgr-09-02*	9(63.8-65.7)	I	Westland A×PI550610	2.5-4.7	Wu and Huang, 2008
*Qstsgr-sbi09i*	9(23.57)	I	PI 607900×BTx623	26.9-27.31	Punnuri et al., 2013
*Qstsgr-sbi09ii*	9(22.57)	I	PI 607900×BTx623	3.9	Punnuri et al., 2013
*Qstsgr-sbi09iii*	9(25.57)	I	PI 607900×BTx623	3.1	Punnuri et al., 2013
*Qstsgr sbi09iv*	9(16.28)	I	PI 607900×BTx623	2.5	Punnuri et al., 2013
*VIS-GBK1*	3(0.01)	K	96-4121×Redlan	2.60	Nagaraj et al., 2005
*VIS-GBK2*	3(1.28)	K	96-4121×Redlan	2.72	Nagaraj et al., 2005
*VIS-GBI8*	3(13.76)	I	96-4121×Redlan	3.83	Nagaraj et al., 2005
*VIS-GBK5*	5(29.41)	K	96-4121×Redlan	2.06	Nagaraj et al., 2005
*SPA-I2*	5(15.01-24.94)	I	96-4121×Redlan	2.21-2.31	Nagaraj et al., 2005
*SPA-K2*	5(34.41)	K	96-4121×Redlan	2.22	Nagaraj et al., 2005
*SPA-K3*	5(83.97)	K	96-4121×Redlan	2.18	Nagaraj et al., 2005

Chou and Huang (2010) first found that the expression of thaumatin-like protein (TLP) increased thousands of times after being bitten by wheat aphids, indicating that TLP may be involved in sorghum’s defense response to greenbug (Chou and Huang, 2010). TLP proteins belong to the PR gene family, widely involved in defense and development processes in plants, animals, and fungi. In plants, the TLP protein belongs to the PR-5 gene family (Irfan et al., 2020). TLPs have been found in various plants such as maize, Arabidopsis, barley, moss, and rice (Ernst et al., 2006; Zefeng et al., 2008; Liu et al., 2024). Additionally, Zhang and Huang (2024) analyzed the NAC gene family in sorghum and identified 9 *SbNAC* genes induced by greenbug by comparing susceptible line BTx623 and resistant line PI607900 (Zhang and Huang, 2024). This proves that the NAC transcription factor family also plays a very important role in sorghum defense processes.

#### 4.2.2 Sorghum defensive response to greenbug infestation

Similar to the response of plants to sorghum aphid feeding, plants also take a series of defense measures to cope with the invasion of greenbug. Firstly, the phloem of sorghum will initiate defense responses. Grover et al. (2019) discovered resistance factors in the vascular tissue of resistant materials through the development of a nested association mapping (NAM) population of sorghum, which can shorten the feeding time of greenbug and thus have resistance response to greenbug (Grover et al., 2019). Harris-Shultz et al. (2019) identified a wax mutant, *bloomless2* (*bm2*), in sorghum that is resistant to greenbug. They studied the differences in resistance of *bm2* mutants to greenbug in five different backgrounds (Tx7078, P898012, P954035, BN109, PI257599) and found that sorghum had the best resistance to greenbug in the background of P898012, and moderate resistance performance in the genetic backgrounds of Tx7078 and P954035 (Harris-Shultz et al., 2019). In addition, hormones, such as SA, JA, and abscisic acid (ABA), also play a crucial role/have function in the response of sorghum to aphid stress (Zhu-Salzman et al., 2004). Zhu-Salzman et al. (2004) proved that treating at the sorghum seedlings stage (growing for 7 days) with methyl jasmonate (Me-JA) can effectively resist greenbug infestation, indicating that JA and its derivatives may play an important role in defending against greenbug (Zhu-Salzman et al., 2004). In addition to the above hormones, Park et al. (2006) found that IAA and gibberellic acid (GA) are also involved in sorghum’s resistance response to greenbug. After 72 hours of aphid feeding, the auxin-induced protein (AIP) began to be upregulated, and the gibberellin-induced protein (GIP) began to be induced (Park et al., 2006).

## 5 Aphids control

### 5.1 Chemical and biological control of sorghum aphid

Aphids, as one of the most serious threats to global sorghum production, pose a risk throughout the entire growth period of sorghum, from seedling to maturity (Xoconostle-Cázares et al., 2023). Chemical control involves the use of insecticides to reduce the populations of aphids affecting sorghum. Currently, commonly utilized insecticides include thiamethoxam, flupyradifurone, and sulfoxaflor (Barbosa et al., 2017; Colares et al., 2017). Research has demonstrated that these insecticides significantly improved efficacy and are approved for large-scale field application (Szczepaniec and Protection, A.J.C, 2018). However, it is essential to exercise caution when using broad-spectrum insecticides, particularly pyrethroids, carbamates, and organophosphates, as their use may unintentionally harm the natural enemies of sorghum aphids. This could potentially lead to outbreaks of secondary pest populations (Catchot, 2015).

In recent years, considering the impact on plants and the environment, researchers have been gradually developing new insecticides that are gentler on plants and more environmentally friendly. Sotelo-Leyva et al. (2023) discovered that extracts of Xanthotoxin from [*Conyza canadensis* (L.) Cronq] have a significant inhibitory effect on sorghum aphids, showing stronger aphid-killing activity at low concentrations (Sotelo-Leyva et al., 2023). Previous studies found that exogenous spraying of a 1‰ concentration of naringenin and rosmarinic acid can effectively enhance sorghum’s resistance to aphids (Zhao et al., 2024b). However, the above-mentioned methods still present risks of inducing plant mutations and environmental pollution, not aligning with current and future development directions.

As the long-term use of chemical pesticides has increasingly impacted humanity, biological control has gradually become a sought-after direction. One of the most common forms of biological control is the utilization of natural enemies of aphids, including parasitoids, parasitic wasps, pathogens, and predators (Hewlett et al., 2019). For instance, in the ecosystem of aphids, ladybugs, lacewings, and *dragonfly larvae* (Order Odonata: Family Aeshnidae) effectively reduce aphid populations through predation. Both *Coccinella septempunctata L*, (Coleoptera: Coccinellidae) and *Harmonia axyridis Pallas*, (Coleoptera: Coccinellidae) ladybug species significantly reduced aphid populations at low density levels (Hewlett et al., 2019). In addition to natural predation, methods for biologically controlling sorghum aphids also include the application of pathogens and parasitoids. For example, research by White (2001) demonstrated that the *pathogen Verticillium lecanii* and the *parasitoid wasp Lysiphlebus testaceipes Cresson* jointly mediate and control the sorghum aphid population in Florida (White, 2001). Although this research has only been conducted at the laboratory stage, it holds potential for large-scale field application in the future.

### 5.2 Chemical and biological control of greenbug

Greenbug use needle-like, piercing mouthparts to extract plant sap from the phloem of their host. While feeding, greenbug inject saliva into the plant to enhance nutrient absorption. The feeding of these greenbugs causes significant damage to wheat and sorghum. In the Great Plains region of the United States, the economic losses caused by greenbug feeding on cereal crops can exceed 100 million dollars annually (Webster, 2000; Giles et al., 2008).

Over the past 50 years, many insecticides have been used to combat greenbug in sorghum fields, mainly categorized into four groups: broad-spectrum carbamates, organophosphates, pyrethroids, and neonicotinoids (Bauernfeind, 1983; Royer et al., 2011). However, with the widespread use of insecticides, people have gradually found that aphids have developed resistance to insecticides. Shufran et al. (1997) found that specific biotypes of greenbug have developed cross-resistance to carbamates and organophosphates (Shufran et al., 1997). Furthermore, with the extensive use of organophosphates, plants have gradually developed necrotic lesions, known as sorghum organophosphate sensitivity (OPS) (Jing et al., 2021).

Here is a diverse range of natural enemies of greenbug, including *ladybugs* (Coleoptera: Coccinellidae), *lacewings* (Neuroptera: Chrysopidae), *parasitic wasps* (Hymenoptera: Aphidiidae, Braconidae), *hoverflies* (Diptera: Syrphidae), and spiders (Araneae), all of which can prey on aphids on sorghum in large numbers (Walker et al., 1973; Gilstrap et al., 1984). Previous studies showed that the release of *L. testaceipes* can served as an effective strategy to prevent greenbug outbreaks in sorghum (Jackson et al., 1970; Gilstrap et al., 1984). With further research and development of biological control methods, we can expect to achieve more sustainable and eco-friendly pest management strategies in agricultural production. This not only helps reduce reliance on chemical pesticides but also protects the health and balance of ecosystems.

## 6 Conclusion and perspectives

**Figure 2:**
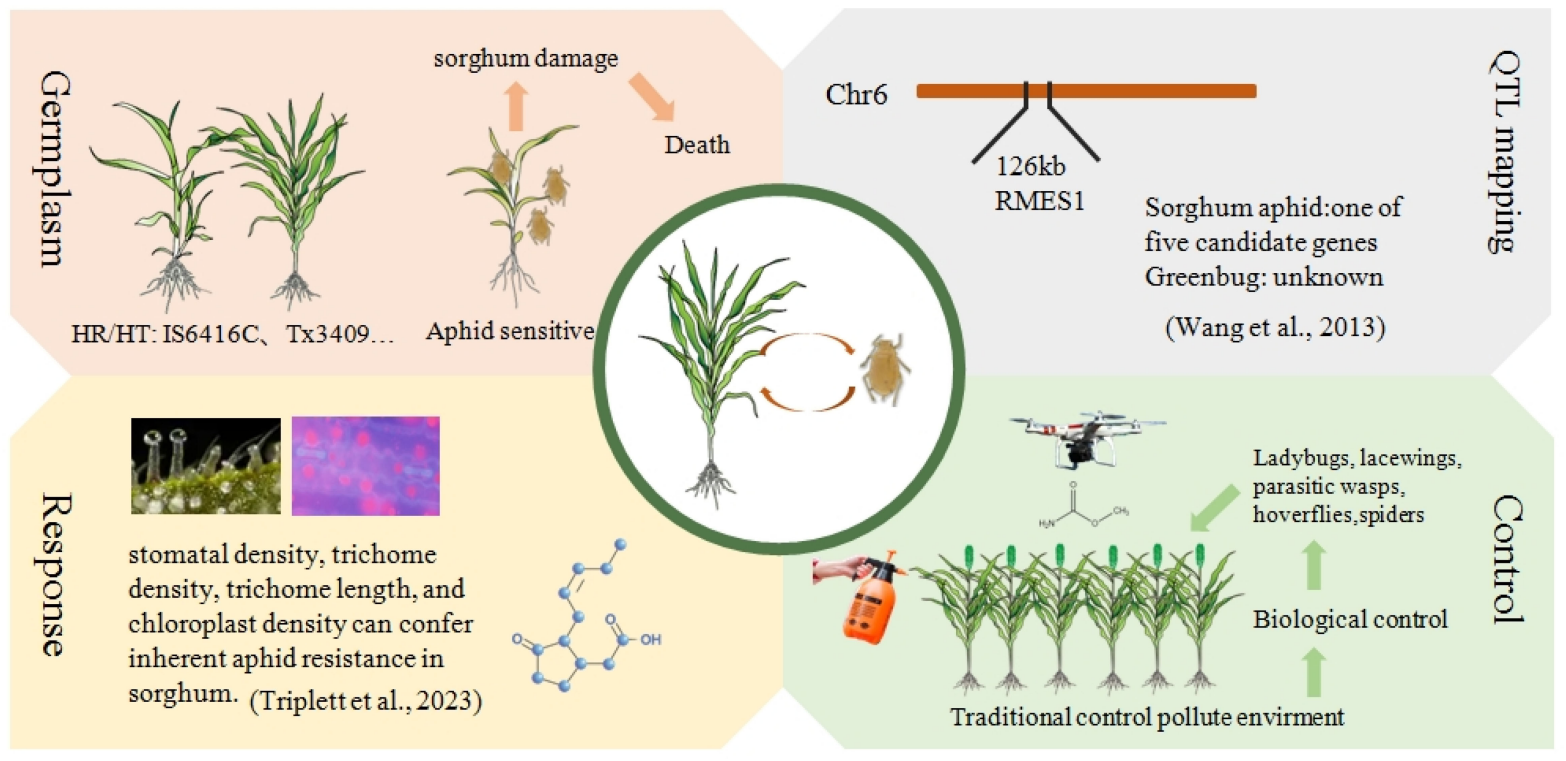
Strategies for enhancing aphid resistance in sorghum. Based on the selection of aphid-resistant materials, effective aphid-resistant lines can be bred. High-throughput sequencing technology can be employed to identify key genes associated with aphids resistance. Additionally, integrating chemical and biological control measures can help manage aphids populations. By utilizing these technologies and methods, the resistance and yield of sorghum can be enhanced.

Aphids, as a significant threat to global crop production, have long been a subject of interest for scientists. Given the serious impact of aphids on sorghum production, breeding aphid-resistant varieties has become a primary goal for crop breeders, despite the challenges involved. Currently, researchers have identified aphid-resistant genes in sorghum within a 126KB region on chromosome 6, with the *RMES1* gene’s specific location being crucial for understanding sorghum’s response to aphid feeding. However, compared with the previous research by Xie et al. (2019), which determined that the tannin 1 gene is the major locus controlling the feeding behavior of sorghum birds and revealed its bird–plant mechanism (Xie et al., 2019). More research is still needed on the aphid resistance of sorghum. Recent studies on aphid-resistant varieties have deepened efforts to mitigate the global agricultural impact of aphids. When introducing foreign varieties for aphid resistance breeding, caution is necessary to address potential risks associated with new hybrids. Notably, sorghum hormone levels change after aphid feeding, offering valuable insights into hormone roles in sorghum’s response to biotic stress. Additionally, research on physiological characteristics like stomatal density, trichome density, trichome length, chloroplast density, leaf thickness, and epidermal cell regularity provides new perspectives on sorghum’s aphid resistance mechanism. On the other hand, the extensive use of pesticides and insecticides can cause soil pollution and enhance the drug resistance of sorghum and aphids. Therefore, the dosage of drugs needs to be increased, creating a vicious cycle. This poses challenges for future research on aphid resistance genes and field management. Therefore, to solve this problem, it is necessary to explore green biological control methods, such as effectively using natural enemies to control aphids (**Figure 2**).

In conclusion, sorghum, as one of the world’s top five crops, holds undeniable importance. However, so far, the aphid resistance genes of sorghum have not been located. Therefore, the aphid resistance genes cannot be applied to breeding research. This is also the direction that future research needs to strive for. Moving forward, greater emphasis should be placed on utilizing biological tools, conducting in-depth research on aphid-sorghum interaction mechanisms, developing more markers to facilitate breeding of aphid-resistant sorghum varieties, and striving to boost global sorghum production.
